# Age-related decline in motion contrast sensitivity due to lower absorption rate of cones and calculation efficiency

**DOI:** 10.1038/s41598-020-73322-7

**Published:** 2020-10-05

**Authors:** Asma Braham chaouche, Daphné Silvestre, Arthur Trognon, Angelo Arleo, Rémy Allard

**Affiliations:** 1 INSERM, CNRS, Insititut de la Vision, Sorbonne Université, 17 Rue Moreau, 75012 Paris, France; 2 Laboratoire Psychophysique de la Vision, École d’optométrie, Université de Montréal, Montréal, QC Canada

**Keywords:** Sensory processing, Motion detection

## Abstract

Motion perception is affected by healthy aging, which impairs the ability of older adults to perform some daily activities such as driving. The current study investigated the underlying causes of age-related motion contrast sensitivity losses by using an equivalent noise paradigm to decompose motion contrast sensitivity into calculation efficiency, the temporal modulation transfer function (i.e., temporal blur) and 3 sources of internal noise: stochastic absorption of photons by photoreceptors (i.e., photon noise), neural noise occurring at the retinal level (i.e., early noise) and at the cortical level (i.e., late noise). These sources of internal noise can be disentangled because there impacts on motion contrast sensitivity vary differently as a function of luminance intensity. The impact of healthy aging on these factors was evaluated by measuring motion contrast sensitivity of young and older healthy adults at different luminance intensities, temporal frequencies and with/without external noise. The older adults were found to have higher photon noise, which suggests a lower photon absorption rate of cones. When roughly equating the amount of photons being absorbed by the photoreceptors, older adults had lower calculation efficiencies, but no significant aging effect was found on temporal modulation transfer function, early noise and late noise.

## Introduction

Motion perception is essential for many daily activities such as walking in a crowded environment, practicing a sport, crossing a street and driving. However, motion perception decline with healthy aging^1–5^, which can reduce the ability of older adults to perform efficiently and safely many daily activities. For instance, motion perception is obviously essential for driving and older adults were found to be less sensitive to changes of vehicle velocities^6^, have difficulties judging vehicle trajectories^7^ and miss judging upcoming collision events^8^, which affects their ability to drive safely. Furthermore, correlations were found that age-related decline in motion perception correlates with age-related decline in driving^9–11^.

A measure of motion perception is motion contrast sensitivity, which is affected with aging at all temporal frequencies in central vision^1,2,5,12^. Although many studies show that healthy aging affects motion contrast sensitivity, the underlying causes for age-related motion contrast sensitivity loss is still debated in the literature. In the early stages of visual information processing, optical changes occur with aging such as lens clouding and miosis, however, studies agree that these changes cannot account for motion contrast sensitivity decline as a more uniform impairment of performance would be expected^3,13^. At the retinal level, no evidence of major changes with aging were found, as the density of cones and ganglion cells are stable through lifespan and neurons in the LGN are also well preserved with aging^13^. However, at a later processing stage, neural changes occur with aging such as a significant degradation of myelinated fibers and synapses in V1 (studies on senescent monkeys^14,15^), more neural noise in extrastriate areas^16^ and impaired inhibitory processes^17^. In sum, motion contrast sensitivity loss with aging may be accounted for physiological changes at the cortical level rather than at the retinal level^4^. The purpose of the current study was to investigate the underlying causes of age-related motion contrast sensitivity losses using a novel psychophysical paradigm that can estimate the impact of various internal noise sources.

Older observers could be less sensitive to motion perception because of a deterioration of the visual information (e.g., more spontaneous neural activity within the visual system) or because they are less efficient at selecting and integrating the relevant information (e.g., lower spatiotemporal summation or more spatiotemporal uncertainty)^18,19^. To test these hypotheses, the current study used an equivalent noise paradigm^20,21^ to factorize motion contrast sensitivity into equivalent input noise, which is a measure of the impact of the internal noise on motion contrast sensitivity, and calculation efficiency, which is related to the signal-to-noise ratio required to detect the signal. In other words, this paradigm was used to determine whether age-related motion contrast sensitivity losses are due to a degraded transmission of the visual signal (equivalent input noise) or a decrease in the ability of the visual system to detect a motion signal in noise (calculation efficiency).

**Figure 1:**
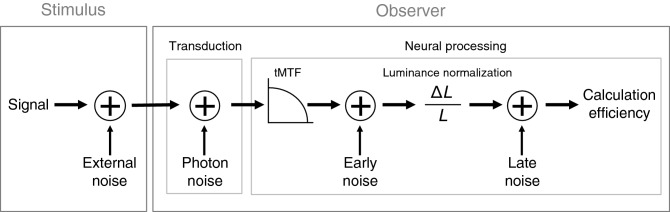
Model comprising photon noise, tMTF, early noise, late noise and calculation efficiency. Photon noise represents the fluctuations of phototransduction. The tMTF models the contrast gain as a function of the temporal frequency due to temporal blur modulating the relative impact of neural noise. Early and late noise represent internal noise occurring before and after luminance normalization.

Given that there is internal noise at all processing stages within the visual system, including stochastic absorption of photons by photoreceptors and neural noise such as spontaneous neural activity, the current study investigated more precisely the impact of aging on various sources of internal noise, compared to studies that have so far only investigated internal noise as a whole^22,23^. To do so, we used the internal noise paradigm^24,25^ enabling to break down the equivalent input noise into the temporal modulation transfer function (tMTF, which is caused by temporal blur reducing the effective contrast of the signal particularly at high temporal frequencies) and three sources of internal noise: stochastic absorption of photons by photoreceptors (i.e., photon noise), neural noise occurring at the retinal level (i.e., early noise) and neural noise occurring at the cortical level (i.e., late noise). This psychophysical paradigm can quantify the impact of these different sources of noise based on two principles^24^: first, sensitivity is noticeably affected only by the greatest noise source, the impact of the weaker noise sources being negligible, and second, the impact of different sources of noise varies differently as a function of luminance intensity. The current study therefore evaluated the impact of healthy aging on various factors (photon noise, tMTF, early noise, late noise and calculation efficiency; Fig. 1) by measuring the motion contrast sensitivity for young and older healthy observers at different luminance intensities, temporal frequencies and with/without external noise.

## Experiment 1

### Methods

#### Observers

The first experiment was conducted on twenty young adults (mean age = 26.5 years, SD = 3.79 years) and twenty-two older adults (mean age = 75.7 years, SD = 4.23 years) from the Silversight cohort (Institut de la Vision, Paris). These participants were selected based on the following inclusion criteria: they had no ocular pathology (e.g., glaucoma, amblyopia, strabismus), no cognitive, neurobiological or vestibular disorders and participants were required to have a good visual acuity ($\ge$ 6/7.5, i.e., $\ge$ 0.8 in decimal units) with their dominant eye. The screening of the participants was undertaken by an otorhinolaryngologist, a neuropsychologist and an ophthalmologist. No participants were under any medication known to alter visual perception,such as benzodiazepine^26^. For three older adults, one of the three noise sources had a negligible impact under all temporal frequencies and luminance intensities, so its impact could not be quantified. Therefore, the results of these three participants were not included in the analyses.

The visual correction of each participant was measured by an orthoptist before the experiment. The participants were wearing trial frames with trial lenses corresponding to their optimal correction for the testing distance to the screen (2 m).

Ethical approval was obtained from the Comité de Protection des Personnes Ile de France V in accordance with the Code of Ethics of the World Medical Association (Declaration of Helsinki), the clinical screening was carried out under medical supervision at the Clinical Investigation Center of the Quinze-Vingts Hospital, Paris, and informed consents of the participants were obtained.

#### Apparatus

Stimuli were presented on a 22.5-inch VIEWPixx LCD designed for psychophysical measurements. The refresh rate of the display was 120 Hz and the mean luminance intensity was 50 cd/m$^2$. At the viewing distance of 2 m, the spatial resolution of the screen was 128 pixels/degree of visual angle. The screen was the only source of light in the room. The Noisy-bit method^27^ implemented independently with each color gun made the 8-bit display perceptually equivalent to an analogue display having a continuous luminance resolution. The output intensity of each color gun was carefully linearized using a Minolta spectroradiometer CS-1000.

#### Stimuli and procedure

Observers were asked to discriminate the drifting direction (left or right) of a sine wave grating by pressing one of two keys. Note that for such a direction discrimination task, the same contrast threshold is typically observed for a detection task, which suggests that detection is based on detectors labeled for direction^28,29^ as in the current study. Since the direction discrimination task was equivalent to a detection task, we defined contrast sensitivity as the inverse of the contrast threshold in absence of noise. Auditory feedback was provided after each answer.

Motion contrast threshold was measured by presenting on a screen a drifting stimulus in the absence and presence of noise at different temporal frequencies (0.9375, 1.875, 3.75, 7.5, 15, and 30 Hz) and at different luminance intensities (from 0.35 to 353 Td) using neutral density filters. The spatial frequency of the grating was fixed at 0.5 cpd.

Stimuli were presented for 500 ms plus an onset and offset ramp (half-cosine) of 250 ms resulting in a total duration of 1 s. The spatial window was a circular aperture of 4 degrees of visual angle plus a smooth edge following a half-cosine of 0.5 degrees of visual angle. Stimuli were presented in the center of an 8.4 $\times$ 8.4 degree of visual angle grey square and were observed by the dominant eye through a 3-mm artificial pupil placed on the trial frame.

The contrast thresholds were measured on a uniform grey background (condition without external noise) and on a noisy background (condition with external noise). The external noise added to the stimulus was a truncated filtered noise^30^ with a low-pass filter with a cutoff frequency of 2 cpd, temporally white (refreshed at 120 Hz), truncated at 1 standard deviation and set at 50% contrast. The energy of the noise was 323 $\mu$s deg$^2$. To avoid triggering a processing strategy shift^31–33^, the noise was continuously displayed (presented during and between trials) and covered the entire screen (i.e., spatiotemporally extended). An underlying assumption of the equivalent noise paradigm used in the current study, is that the effective calculation efficiency in absence of noise is the same as the calculation efficiency measured in high noise. Although the equivalent noise paradigm is generally used with white noise^20,21,34,35^, some argue that white noise may cause neural activity over a wide range of frequency channels, which could interfere with the calculation efficiency in high noise (e.g., through lateral inhibition^36,37^), but not with the effective calculation efficiency in low noise. However, the fact the calculation efficiency measured in high noise (i.e., when the external noise level is strong enough to considerably affect performance) is typically the same at all levels of high noise (i.e., slope of 1 in log-log units) strongly suggests that the measured calculation efficiency is independent of the level of activation in the various frequency channels, and many recent studies^28,31^ rather argue that external noise should be analogous to internal noise, that is, spatially, temporally and spectrally widely distributed, as in the current study. Furthermore, cross-channel suppression would not affect apparent contrast or contrast threshold in high noise^28^ we therefore are not aware of any direct evidence that cross-channel suppression noticeably affect the assessment of calculation efficiency and the equivalent noise paradigm was implemented in the standard way^20,21,38^, that is, with white noise.

To evaluate calculation efficiency, contrast thresholds were measured in the presence of high external noise^21^. Since early temporal filtering can considerably bias the measurement of the calculation efficiency at very high temporal frequencies^39^ (i.e., 30 Hz in the current study) and because it was not possible to estimate the calculation efficiency at 30 Hz (contrast thresholds in noise of the older adults were to high and its measurement was not reliable), the calculation efficiency was measured up to 15 Hz and it was estimated at 30 Hz based on an extrapolation from the calculation efficiencies at lower temporal frequencies^25^. Note that early temporal filtering can nevertheless modestly bias calculation efficiency at lower temporal frequencies^39^ and this bias varies with luminance intensity^40^, but is minimized at higher luminance intensities^25^. Given that almost all the variation of sensitivity with temporal frequency is due to the equivalent input noise rather than calculation efficiency^39^ and that it is not possible to avoid such biases, the calculation efficiency was assessed at the highest luminance intensity and the modest potential biases in the measurement of the calculation efficiency were considered negligible in this first experiment. Nevertheless, the second experiment will provide a control that directly assess these potential biases. Thus, contrast thresholds in high noise that enable the estimation of the calculation efficiency was measured to temporal frequencies up to 15 Hz at the highest luminance intensity (353 Td) and was considered to be independent of luminance intensity^25^.

Contrast thresholds in the absence of external noise were measured at different luminance intensities and temporal frequencies, in order to discern three sources of internal noise and the tMTF. To do this, the conditions (temporal frequency and luminance intensity) were selected to probe these components of the model based on a previous study^25^ that evaluated the sources of noise limiting motion contrast sensitivity over a wide range of temporal frequencies and luminance intensities. Late noise affects motion contrast sensitivity at high luminance intensities and its impact varies with temporal frequency^25,39^, so contrast thresholds were measured at all temporal frequencies (0.9375 to 30 Hz) at the highest possible luminance intensity (353 Td). Photon noise affects motion contrast sensitivity at low luminance intensities and low temporal frequencies, so contrast thresholds were measured at 0.9375, 1.875 and 3.75 Hz for which the observer had a retinal illuminance of 1.1, 3.5 and 11 Td, respectively, obtained by adding neutral density filters placed on the trial frame worn by the observer. Since early noise is assumed to be temporally white (i.e., spontaneous neural activity is not expected to be correlated over time), but its impact is modulated by the tMTF, the tMTF can be estimated by measuring the impact of the early noise at various temporal frequencies^25^. To enable the estimation of the tMTF, early noise was measured at 3.75, 7.5, 15 and 30 Hz, values for which the retinal illuminance was set to 0.35, 3.5, 35 and 110 Td, respectively.

Contrast thresholds were measured using a 3down1up staircase procedure^41^ in which the contrast of the stimulus increased or decreased depending on the correctness of the observer’s response with a step size of 1.25. The staircase was interrupted after 14 inversions and the contrast threshold was estimated as the geometric mean of the last 10 inversions. Such a staircase converged to a criterion level of 79% correct response. Two staircases were performed for each condition. The contrast threshold for a given condition was estimated as the geometric mean of the two staircases.

The experiment was conducted in two sessions of approximately 2 h each. For each participant, the sessions were performed approximately 1 week apart and the order of the sessions was randomized across the participants. In one session, contrast thresholds were measured at the highest luminance intensity and in the other session they were measured at low luminance intensities. For the latter, the observers were light-adapted for 30 min by wearing on the trial frames a neutral optical density filter of 3. For each session, the order of the temporal frequencies was randomized and the two staircases of a given condition (one temporal frequency and one luminance intensity) were measured in the same luminance intensity block to light adapt the observers only once. For the session at the highest luminance intensity (i.e., 353 Td), two contrast thresholds were measured for each temporal frequency to estimate the equivalent input noise and the calculation efficiency: one in absence of noise and the other in high noise (except at 30 Hz where the contrast threshold of the older adults could not be measured because they were not sensitive enough to perceive the signal at the maximum contrast). For the other session, the participants adapted for the lowest luminance intensity (0.35 Td) and were then tested at 0.35, 1.1, 3.5, 11, 35 and 110 Td, in that order.

The calculation efficiency (*k*(*F*)) was estimated from Eq. (1) where *E* is the energy threshold, which is proportional to the squared contrast threshold^21,23^:

1 $$\begin{aligned} k(F) = \frac{(d' +\sqrt{0.5})^2N_{ext}}{E(N_{ext},L,F)-E(0,L,F)} \end{aligned}$$

where $E({N}_{ext},{L,F)}$ is the energy threshold in high noise, *E*(0, *L*, *F*) the energy thresholds in absence of noise, $N_{ext}$ the external noise energy (323 $\mu$s deg$^2$) and ${d}' =1.16$.

The estimated calculation efficiencies as a function of temporal frequency of each observer were fitted with quadratic functions and these fits (i.e., ${k}_{fit} {(F)}$) were then used to estimate the calculation efficiency at each relevant temporal frequency (0.9375 to 30 Hz) and the equivalent input noise at the various luminance intensities:

2 $$\begin{aligned} N_{eq}(L,F) = \frac{k_{fit}(F)}{(d' +\sqrt{0.5})^2}E(0,L,F) \end{aligned}$$

The yellowing of the lens, occurring with healthy aging, filters out primarily the short wavelengths, which reduces the retinal illuminance. To evaluate its potential impact on contrast thresholds, a controlled condition was tested in which stimuli were presented on a red background (i.e., red color gun projecting wavelength longer than 580 nm). Indeed, the long wavelengths (red) are little affected by the yellowing of the lens that filters mainly shorter wavelengths^42^. Therefore, if the age-related yellowing of the lens had a considerable impact on contrast thresholds with gray stimuli, then the age-related sensitivity loss would be much reduced with red stimuli for which the effect of lens yellowing should be negligible. These control conditions were performed at 35 Td and 15 Hz, at which the observer was limited by early noise^25^, and at 3.5 Td (by adding an optical density filter of 1) and 1.88 Hz, at which the observer was limited by photon noise^25^. These controlled conditions were tested in the dark-adaptation session at the same time as their respective luminance intensity block (i.e., 3.5 and 35 Td).

#### Model

The recently developed model^24,25^ was applied on the estimated equivalent input noise of each observer (${N}_{eq} {(L,F)}$) at the different luminance intensities (*L*) and temporal frequencies (*F*), which made it possible to estimate the tMTF and the three sources of internal noise according to the following equation:

3 $$\begin{aligned} N_{eq,fit}(L,F) = \frac{N_{photon}}{L}+\frac{1}{tMTF^2(F)}\biggl (\frac{N_{early}\times N_{photon}^2}{L^2}+N_{late}(F)\biggr ) \end{aligned}$$

where ${N}_{photon}$ is photon noise, ${N}_{early}$ is early noise, and ${N}_{late}$ is late noise.

This model has five degrees of freedom: one for the photon noise (constant with respect to temporal frequency), one for the early noise (constant with respect to temporal frequency), two for the late noise (linear function with respect to temporal frequency), and one for tMTF^25,43^.

The five free parameters of the model were estimated for each participant and then the mean was evaluated to obtain the five parameters for each group (young and older adults).

### Results

#### Motion contrast sensitivity

**Figure 2:**
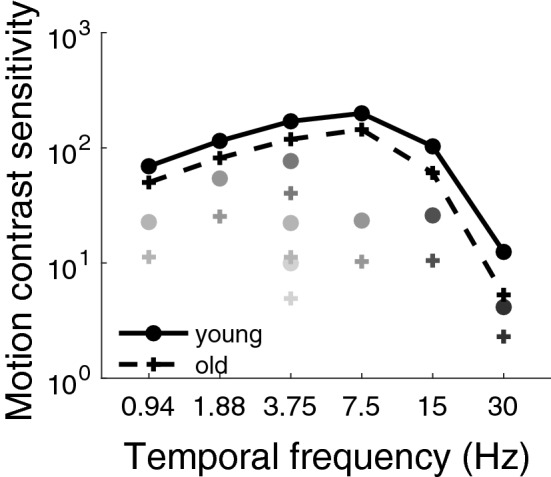
Motion contrast sensitivity of young (circles) and older (crosses) observers. The lightest gray from the darkest gray represents the different luminance conditions: 0.35, 1.1, 3.5, 11, 35, 110 and 353 Td, respectively. Motion contrast sensitivity at the highest luminance intensity (i.e., 353 Td) was measured at all temporal frequencies (from 0.94 to 30 Hz), so for clarity the markers were connected with lines for the young adults and dashed lines for the older adults. The error bars represent the standard error of the mean (most of them are not visible because they are smaller than the symbol size).

Motion contrast sensitivity functions at different luminance intensities for young and older adults are represented in Fig. 2. At the highest luminance intensity, the motion contrast sensitivity functions followed the typical band-pass function peaking around 8 Hz^44,45^. Older adults were less sensitive than young adults at all temporal frequencies and all luminance intensities with a factor ranging from 1.4 to 2.5 (in contrast units). A greater age-related decline was found at low luminance intensities, where all the younger adults had a greater sensitivity than the mean sensitivity of the older adults.

Since the participants performed the task in two separate sessions (order of the sessions was randomized between the participants) a learning effect could have occurred over time, resulting in a better performance in the last session. A three-way analysis of variance (ANOVA; order of sessions $\times$ age $\times$ condition) did not show any significant effect of the sessions’ order (${F}(1,34)=0.66$, ${p}=0.42$), suggesting that no considerable learning effect occurred between the two sessions. Individual differences such as gender were assessed with a three-way ANOVA (gender $\times$ age $\times$ condition), which showed no significant effect of gender (${F}(1,29)=0.63\,{p}=0.44$), suggesting that men and women had similar motion contrast sensitivity.

#### Calculation efficiency

**Figure 3:**
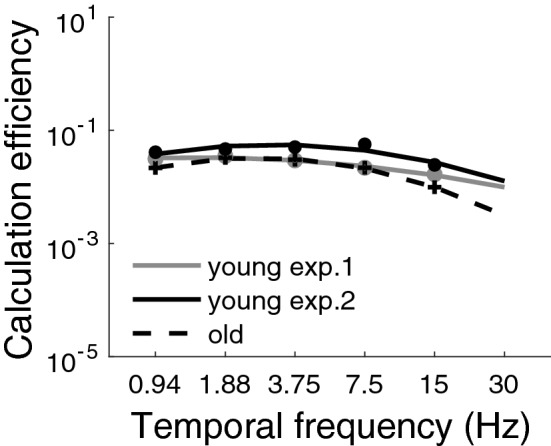
Calculation efficiency as a function of temporal frequency at 353 Td for the young observers (grey circles, from experiment 1), for the older observers (black crosses, from experiment 1) and for the young observers at 110 Td (black circles, from experiment 2). The solid lines (young adults) and dashed lines (older adults) represent the fits (i.e., quadratic functions). The error bars represent the standard error of the mean (most of them are not visible because they are smaller than the symbol size).

**Figure 4:**
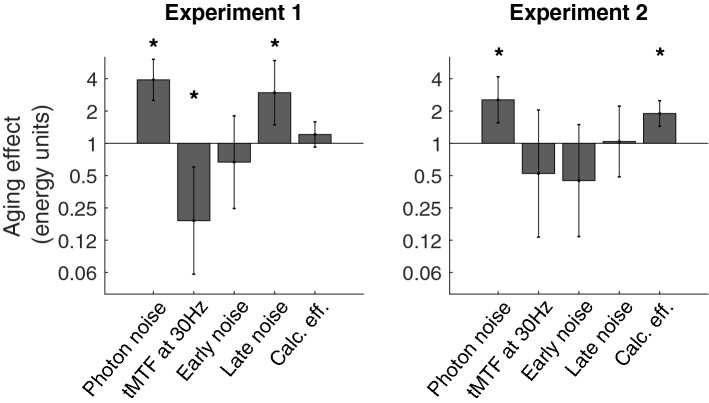
The aging effect on the different parameters of the model. An aging effect greater than 1 for photon noise and late noise (i.e., mean of late noise) represents more noise for the older adults. An aging effect smaller than 1 for early noise represents less noise for the older adults and for tMTF it represents a tMTF that is less low-pass for the older adults. An aging effect greater than 1 for calculation efficiency represents older observers being less efficient than the young observers. The error bars represent a 95% confidence interval (± 1.96 $\times$ the standard error of the difference) and the asterisks represent a significant aging effect $(p<0.05)$.

The calculation efficiency for young and older adults at high luminance intensity (353 Td) is shown in Fig. 3. The mean aging effect across temporal frequency was of a factor 1.2 in energy units, reported in the left graph of Fig. 4. A two-way analysis of variance (ANOVA; age $\times$ temporal frequency) did not show any significant effect of age (${F}(1,37)=1.87$, ${p}=0.18$), but a significant effect of temporal frequency (${F}(4,148)=43.8$, ${p}<0.001$). A significant interaction between age and temporal frequency, (${F}(4,148)=4.64$, ${p}<0.01$) was found. To better understand the interaction between age and temporal frequency, Tukey’s post-hoc tests were performed, but no statistical difference were observed at any temporal frequency.

Note that at 30 Hz, contrast threshold in noise was not measured, but was extrapolated from the fits, however this extrapolated data was not taken into account in the statistical analysis of calculation efficiency. The data was fitted with the statistically best function, which was a quadratic function.

#### Equivalent input noise

**Figure 5:**
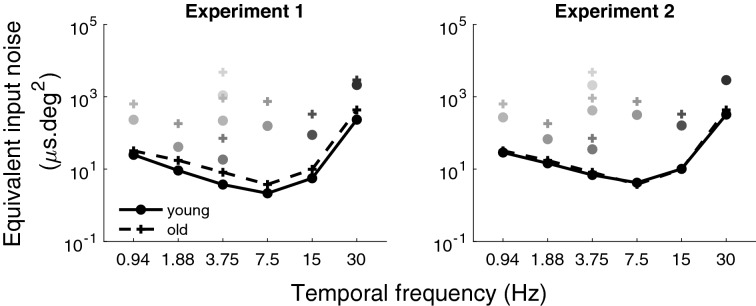
The equivalent input noise. The equivalent input noises for the young observers (circles) and for the older observers (crosses) from experiment 1 (graph on the left) and from experiment 2 (graph on the right). The lightest gray from the darkest gray represents the different luminance conditions: 0.35, 1.1, 3.5, 11, 35, 110 and 353 Td, respectively. The data for the highest luminance intensity (i.e., 353 Td) were connected for clarity, with lines for the young adults and dashed lines for the older adults, as it was the only luminance condition where all temporal frequencies (0.94 to 30 Hz) were measured. The error bars represent the standard error of the mean (most of them are not visible because they are smaller than the symbol size).

Using Eq. (2) (see Methods section), the equivalent input noise for young and older adults (left graph of Fig. 5), at different luminance intensities and different temporal frequencies, was derived from the motion contrast sensitivity measured in the absence of noise (Fig. 2) and the fits of the calculation efficiency from the calculation efficiencies estimated at the highest luminance intensity (Fig. 3), which was assumed to be independent of luminance intensity. Equivalent input noise was generally greater for the older observers, especially at low luminance intensities.

#### Internal noise sources

**Figure 6:**
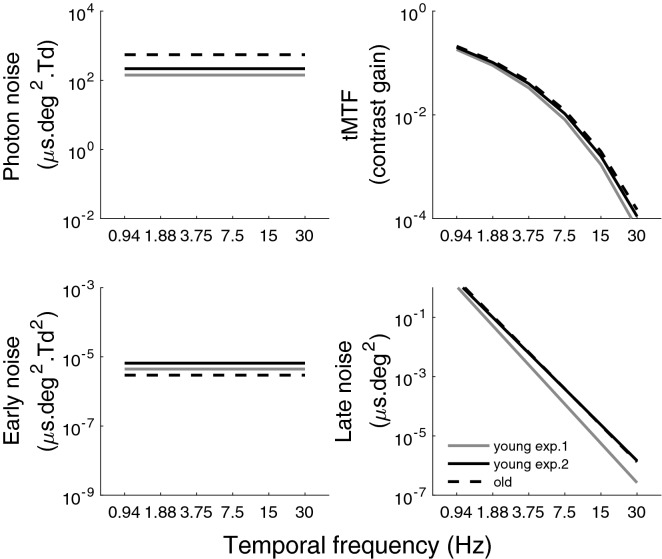
Sources of internal noise. The top left graph represents the photon noise as a function of temporal frequency (independent of temporal frequency). The top right graph represents the fitted tMTF. The bottom left graph represents the early noise (independent of temporal frequency). The bottom right graph represents the late noise as a function of temporal frequency (linear function). The mean fit of our model is represented by grey solid lines for the young observers in experiment 1, black dash lines for older observers (experiment 1) and black solid lines for the young observers in experiment 2.

Fitting the observer’s model for each participant (Fig. 1; Eq. (3)) based on the equivalent input noise derived at different temporal frequencies and luminance intensities enabled us to decompose the equivalent input noise (left graph of Fig. 5) into photon noise, early noise, late noise and tMTF. The impact of these three sources of internal noise as well as temporal integration as a function of temporal frequency are represented in Fig. 6 for young and older adults. The aging effects on these parameters are summarized on the left graph of Fig. 4.

Photon noise, which had only one degree of freedom as it was assumed to be temporally white (top left graph of Fig. 6), was significantly affected with aging (${t}(37)=6.11$, ${p}<0.01$). More specifically, the effect of age on photon noise induced a decrease in sensitivity of a factor of 3.89 in energy units. This result suggests that photoreceptors of the older adults absorbed 3.89 times less photons than the ones of young adults.

The tMTF was also modeled by a single parameter representing the steepness of the low-pass temporal function (top right graph of Fig. 6). A significant aging effect was also found ($t(37)=-\,2.78$, $p<0.01$). Surprisingly, however, this result suggests that the visual system of the older observers has less temporal blur. This result was surprising and unexpected given that sensitivity is more affected by aging at higher temporal frequencies (but see experiment 2).

Early noise was assumed to be temporally white (bottom left graph of Fig. 6) and was fitted with a single parameter. No significant aging effect was found for early noise (${t}(37)=-\,0.82$, ${p}=0.42$), which suggests that healthy aging has little impact on early noise.

Late noise (bottom right graph of Fig. 6), which drops with temporal frequency, was fitted by a linear function^25^. Older observers were found to have significantly more late noise on average (i.e., the late noise’s mean across temporal frequency) (${t}(37)=3.09$, ${p}<0.01$) and a trend was observed for the slope of late noise compared to the young observers (${t}(37)=1.93$, ${p}=0.06$). These results suggest that healthy aging has an impact on late noise particularly at high temporal frequencies.

#### Control for the yellowing of the lens

**Figure 7:**
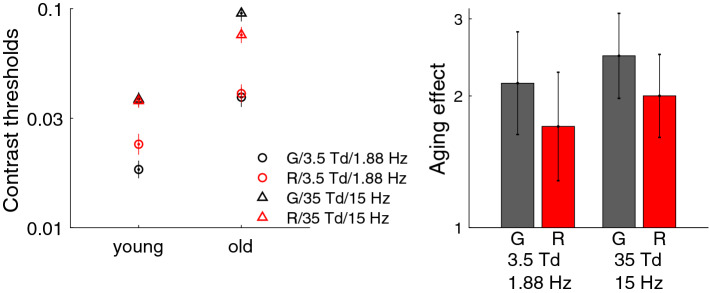
Experimental control of the yellowing of the lens. The graph on the left represents the contrast thresholds of the young and older observers for the 3.5 Td and 1.88 Hz condition (circles), for which photon noise was the dominant source of noise and the 35 Td and 15 Hz condition (triangles), for which early noise was the dominant source of noise. For both conditions, contrast thresholds were measured on a red (R) and grey (G) screen. The error bars represent the standard error of the mean. The graph on the right represents the aging effect in contrast units (contrast thresholds of older/young) for each condition. The error bars represent a 95% confidence interval (± 1.96 $\times$ the standard error of the difference).

Results when controlling for age-related yellowing of the lenses are shown in Fig. 7. A three-way ANOVA (age $\times$ color $\times$ condition) on the contrast thresholds measured for different temporal frequencies and luminance intensities (3.5 Td and 1.88 Hz; 35 Td and 15 Hz) showed a significant effect of age (${F}(1,37)=40.4$, ${p} <0.001$), that is, young observers had, on average, better thresholds than older observers. A significant interaction between age and color (${F}(1,37)=11.1$, ${p}<0.01$) was due to lower aging effects with the red stimuli, suggesting a small effect of the yellowing of the lens occurring with healthy aging. Nevertheless, even when the impact of the yellowing of the lens was negligible, older observers remained considerably less sensitive than young observers for conditions under which photon noise or early noise dominated.

## Experiment 2

In the first experiment, a considerable aging effect was observed on photon noise suggesting that photoreceptors of older observers were absorbing about 3.9 times less photons, which is consistent with a previous study on spatial contrast sensitivity^46^. Given that the tMTF can slightly bias the measurement of calculation efficiency^39^ and this bias varies with luminance intensity^25,40^, a considerably lower absorption rate could have affected the measurement of the calculation efficiency slightly differently for the two age groups. Indeed, although both age groups were tested at the same luminance intensity, the lower absorption rate for older observers implies that their effective luminance intensity (i.e., amount of absorbed photons) was lower. Thus, it is possible that the aging effect observed on calculation efficiency might have been biased by a lower absorption rate of the photoreceptors. Given that the equivalent input noise is derived from the threshold in absence of noise and the calculation efficiency, a bias in the aging effect of the calculation efficiency would equally bias the aging effect on the equivalent input noise and would therefore affect the aging effect on some other parameters derived from the equivalent input noise, such as photon noise, tMTF, early noise and late noise.

The aim of the second experiment was to reevaluate the effect of aging on the various parameters while compensating for a potential bias in the measurement of calculation efficiency due to a lower absorption rate of older observers. Given that older adults absorbed about 3.9 times less photons than the younger group on average (photon noise was 3.9 times greater for the older group in experiment 1), we reevaluated the calculation efficiency (i.e., contrast threshold in high noise) of the young observers while reducing the retinal illuminance by a similar proportion. In other words, calculation efficiency was reevaluated for young observers under conditions in which they absorbed a similar amount of photon as older observers. The reevaluated calculation efficiency in young observers was then combined with the contrast threshold in absence of noise in experiment 1 to derive the equivalent input noise and thereby the various parameters (photon noise, tMTF, early noise and late noise).

### Methods

The same apparatus, stimuli and procedure were used as for experiment 1. The study was conducted on the young participants of the experiment 1, but three of them were not available so only 17 were tested. The aging effect on the photon noise was simulated by adding an optical density filter of 0.5, which reduces the luminance intensity by a factor of 3.16 (from 353 to 110 Td). The new measures of calculation efficiency (*k*(*F*)) were estimated in the same way as in the experiment 1 using Eq. (1) based on the contrast threshold in absence of noise at the highest luminance intensities (*E*(0, *L*, *F*)) obtained in the first experiment, and with the thresholds in noise (${E(}N_{ext}$,*L,F)*) obtained in the second experiment. Note that the thresholds in absence of noise have a negligible impact on the estimated calculation efficiency, which only considerably depends on the threshold in high noise^21^. The exact same analysis was then performed with the new calculation efficiency (*k*(*F*)): a fit with a quadratic function was applied to the calculation efficiency (${k}_{{fit}}{(F)}$), which was used to recalculate the equivalent input noise ($N_{eq}{(L,F)}$, Eq. (2)), which was then used to refit the model (Eq. (3)). As a result, in the second experiment only contrast thresholds were measured in noise at different temporal frequency (0.9375 to 15 Hz). Observers light-adapted for 2 min before the start of the experiment.

### Results

#### Calculation efficiency

Reducing luminance intensity slightly affected the measurement of the calculation efficiency (Fig. 3). As expected from a previous study^40^, reducing luminance intensity counter-intuitively improved performance. Indeed, contrast thresholds were lower (i.e., better) at the lower luminance intensity. Given that calculation efficiency depends slightly on the amount of photons being absorbed and that young and older observers do not absorb the same amount of photons (different photon noises), the age-related effect on calculation efficiency per se should be compared under conditions in which both age groups absorbed similar amount of photons, that is, the calculation efficiency measured under low luminance intensity for the young observers in experiment 2 and the calculation efficiency measured at high luminance intensity for the older observers in experiment 1. The mean aging effect across temporal frequency was of a factor 1.9 in energy units (1.38 in contrast units), reported in the right graph of Fig. 4.

A two-way analysis of variance ANOVA (age $\times$ temporal frequency) on the calculation efficiency showed a significant effect of age (${F}(1,34)=23.4$, ${p} <0.001$, with a very large effect size: Cohen’s d = 1.61), and temporal frequency (${F}(4,136)=44.0$, ${p}<0.001$), as well as a significant interaction between these factors, age and temporal frequency, (${F}(4,136)=4.7$, ${p}<0.01$). Tukey’s post-hoc test on the interaction between age and temporal frequency showed that older adults had a significantly poorer calculation efficiency than young adults at 1, 7.5 and 15 Hz, suggesting that the effect of aging on calculation efficiency varies with temporal frequency, particularly at low and high temporal frequencies.

#### Equivalent input noise

Given that the equivalent input noise was derived from the calculation efficiency and the thresholds in absence of noise, changing the calculation efficiency has an impact on the estimated equivalent input noise. Thus, equivalent input noise needs to be reassessed. As shown in Fig. 5 (right graph), when roughly equating the number of photons absorbed in both age groups by reducing the luminance intensity for young observer, the equivalent input noise for young and older adults were remarkably similar at the highest luminance intensity and a smaller aging effect compared to experiment 1 (left graph of Fig. 5) was observed at low luminance intensities.

#### Internal noise sources

The parameters of the model for young and older adults at different temporal frequencies and luminance intensities are represented in Fig. 6 and summarized in Fig. 4 (right graph). The goodness of the model fitting the equivalent input noise data was estimated with R$^2$, which was 96.4% for the young adults and 95.4% for the older adults, suggesting that the model was a good fit. Photon noise was significantly affected by aging (${t}(34)=3.69$, ${p}<0.001$, with a very large effect size: Cohen’s d=1.23), with older adults having 2.5 times more photon noise than the young adults. This result suggests that in the current study, photoreceptors of the older population absorbed 2.5 times less photons than the ones of the young observers. No significant aging effect was found for tMTF (${t}(34)=-\,0.85$, ${p}=0.40$) or for early noise (${t}(34)=-\,1.34$, ${p}=0.19$). Aging did not significantly affect the mean ($t(34)=0.10$, ${p}=0.92$) and the slope (${t}(34)=-\,0.42$, ${p}=0.67$) of the late noise. Note that given that equivalent input noise at the highest luminance intensity is limited by late noise and no effect of aging was observed in those conditions, it is not surprising that we observe no effect of aging on the parameters affecting equivalent input noise at this high luminance intensity: late noise and the tMTF.

The second experiment shows that equating the amount of photons being absorbed between young and older observers is important when evaluating calculation efficiency, which is therefore also important to derive the equivalent input noise. The first experiment suggests that the older adults had more late noise in average and less temporal blur, but after equating for the amount of photons absorbed (experiment 2), these aging effects were no longer significant (right graph of Fig. 4).

## General discussion

When roughly equating the amount of photons being absorbed by the photoreceptors between the two age groups, the current study found that older adults had more photon noise and a lower calculation efficiency, but no significant age-related effect was found for tMTF, early noise and late noise. These results suggest that healthy aging affects the amount of photon absorbed by photoreceptors and require a greater signal-to-noise ratio to detect the signal, but has no or little impact on the amount of neural noise limiting motion contrast sensitivity within the visual system either at the retinal level (i.e., early noise) or the cortical level (i.e., late noise), and the tMTF (i.e., temporal blur due to the early integration time). So the loss of motion contrast sensitivity with aging would be mainly due to an increase in photon noise and a decrease in calculation efficiency.

The increase in photon noise with aging suggests that photoreceptors in older adults absorbed 2.5 times fewer photons than those in younger adults, which is consistent with a previous study^46^ on spatial contrast sensitivity using an analogous noise paradigm that found an aging effect on photon noise. Two age-related causes are typically cited as reducing the proportion of photons being absorbed by photoreceptors: senile miosis (reduction of the pupil diameter occurring with healthy aging) and yellowing of the lens (tendency to filter the light of short wavelengths). In the current study, the effect of senile miosis was neutralized by the use of an artificial pupil thus, senile miosis had little impact on the photon absorption rate of the photoreceptors. However, controlled conditions revealed that a smaller aging effect was observed when using long wavelengths (red light), that are little affected by the yellowing of the lens, compared to a grey background condition. This result suggests that the yellowing of the lens of the older adults had a small impact on the photon noise estimation, thus the lower photon absorption found for the older adults is partly explained by the yellowing of their lens absorbing a portion of the light (i.e., short wavelengths). The 2.5 aging effect found for the photon noise is therefore explained by the lower absorption rate of the older adults’ photoreceptors and by the yellowing of their lens. The fact that ocular factors cannot explain most of the lower photon absorption rate with aging suggests that the lower absorption rate of the older adults’ photoreceptors could be explained by photoreceptors being less efficient as suggested by Silvestre and colleagues^46^. Indeed, given that there is no considerable loss of cones with healthy aging^47,48^, the fact that less photons are absorbed suggests that the absorption rate of cones is affected with aging. Different physiological changes occurring with aging could be at the origin of a lower absorption rate of photons by cones. For instance, a possible explanation is a morphological change of the cones due to the decreased density of rods (i.e., approximately a 30% loss with aging^48–50^). Indeed, the loss of rods disorganizes the alignment of the cones by giving less support to the cones and therefore induces a change in the orientation of the cones, reducing their absorption rate^51^. Another explanation for the less efficient photoreceptors with aging could be due to a physiological change occurring in the transduction cascade, with for instance more spontaneous noise preventing photon absorption. A previous study^52^ suggested that cone noise originates from fluctuations of the concentration of cGMP (a secondary messenger in phototransduction). Indeed, a high concentration of cGMP inhibits the cone activation. In presence of light, the transformation of cGMP to GMP might be degraded with aging, keeping a concentration of cGMP high enough to not allow the hyperpolarization of the photoreceptor cell, overall having for consequence less activated photoreceptors with aging. Further studies are required to elucidate the physiological cause of a decline in the absorption rate of photons by photoreceptors.

In the current study, neural noise (early noise and late noise) was not affected by aging, which was unexpected because some previous studies suggest that neural noise may increase with aging as there is more spontaneous activity of neurons due to GABA reduction^53^ affecting spatial suppression^54^, or due to degradation of myelinated fibers and synapses in V1^14,15^. However, these age-related neurophysiological alterations are not necessarily inconsistent with the current findings. There are many sources of internal noise within the visual system, but this does not mean that they have a considerable impact on all perceptual tasks. The current study suggests that the dominant internal noise source that limits motion contrast sensitivity is not considerably affected with aging, but this does not imply that other weaker internal noise sources are not affected with aging. For instance, aging considerably affects photon noise, but this noise has a negligible impact on motion contrast sensitivity at high luminance intensities when late noise dominates. Similarly, if there are many sources of late noise, only the dominant one will considerably affect sensitivity. Thus, the current study suggests that the main sources of early and late noise limiting motion contrast sensitivity are not considerably affected with aging, but this does not imply that weaker sources are not considerably affected. Another possibility is that age-related physiological alterations affect the signal and internal noise by the same proportion. For instance, an age-related GABA reduction would increase the neural activity^53^, but if it increases the signal strength and the internal noise by the same proportions, then it would have no impact on the signal-to-noise ratio and thereby no impact on performance. Therefore, previous findings of physiological changes occurring with aging, such as GABA reduction^53^ affecting spatial suppression^54^, are not necessarily incompatible with our current findings that there is no considerable age-related increase in neural noise limiting motion contrast sensitivity.

Interestingly, the current absence of age-related effect on late noise appears to diverge from our previous study on spatial contrast sensitivity^46^, which found an age-related increase in late noise. Although we cannot exclude the possibility that the different results are only due to chance (i.e., aging would actually equally affect the late noise limiting spatial contrast sensitivity and the late noise limiting motion contrast sensitivity), the possibility that aging affects late noise limiting spatial contrast sensitivity without affecting the late noise limiting motion contrast sensitivity is not inconsistent. Indeed, it may not be the same internal noise source limiting these sensitivities, so it is possible for aging to affect one late noise without affecting the other.

The current study found no significant aging effect on tMTF, which were found to be remarkably similar in young and older observers. These results suggest that the temporal properties of cones and early neural processes are relatively unaffected with healthy aging, which is apparently conflicting with a previous study^55^ using electroretinography (ERG) recordings suggesting that healthy aging affects temporal response properties at the photoreceptor and bipolar cell level. Nevertheless, the results from the age-related difference in the temporal response observed in this ERG study and the lack of difference observed in the current psychophysical study are not necessarily incompatible. The age-related ERG difference could be caused by a lower absorption rate for the older population and not to different temporal response per se. Indeed, maybe similar temporal responses would be observed if the quantity of photon absorbed by the two age groups were equated. This explanation would be analogous to the current study, which observed an age-related difference in temporal response when the number of photons absorbed was not equated (experiment 1), but not when it was equated (experiment 2).

The age-related decline in calculation efficiency (when equating for the amount of photons being absorbed) shows that the older adults required a greater signal-to-noise ratio to detect the signal. Indeed, in high external noise, the internal noise has a negligible impact and the contrast threshold only depends on the calculation efficiency. The current results are not sufficient to determine why older observers required a greater signal-to-noise ratio, but we can speculate. This age-related decline in calculation efficiency for motion contrast sensitivity is analogous to the age-related decline in calculation efficiency for spatial contrast sensitivity^46^ and was speculatively attributed to a decline in the ability to integrate spatial information. Analogously, the age-related calculation efficiency decline for motion contrast sensitivity could be due to an impaired spatial or temporal integration process, that is, older adults may focus on a smaller spatial area or may not be able to consider only the moments during the stimulus presentation. Further studies are required to test these hypotheses.

Photon noise was found to be the main factor responsible for age-related motion contrast sensitivity loss in the present study, and for age-related spatial contrast sensitivity loss in a previous study^46^. Given that all visual functions require the absorption of photon by photoreceptors, one could expect a correlation in age-related declines for many visual functions, but the existence of a common factor responsible for the visual perception loss occurring with healthy aging has not been found^13,56–58^. A recent study^57^ found no correlation between age-related declines in a large number of visual tasks (e.g., visual acuity, orientation discrimination, visual search), which suggests that age-related visual perception losses do not have a common factor. However, the development of the internal noise paradigm shows that the absorption rate of photoreceptors does not always have a noticeable impact on visual tasks. A consequence of the lower absorption rate of photons occurring with aging, which is due to smaller pupils (miosis), the yellowing of the lens and to less efficient cones, is a greater difficulty for the older population to perceive motion under dim light, such as when driving at night^59^. At high luminance intensities, however, sensitivity is generally independent of the amount of photon absorbed because it is independent of luminance intensity (Weber’s law), in which case the absorption rate has no significant impact. Consequently, the age-related decline in the absorption rate would have no noticeable effect on many visual tasks, which are generally performed at high luminance intensities. In fact, some findings in the current study are actually consistent with the hypothesis that aging have different impact on different visual tasks: at high luminance intensities, an aging effect was found for late noise limiting spatial contrast sensitivity^46^, but in the current study, no aging effect was found for late noise limiting motion contrast sensitivity. Thus, this may suggest weak correlations between these two visual tasks under the conditions in which the Weber’s law applies, which would be in agreement with the little age-related correlations between various visual functions^57^.

## Conclusion

The current study investigated the underlying cause of age-related motion contrast sensitivity loss. The results suggest that the main causes of age-related motion contrast sensitivity losses is the absorption rate of cones, which affects motion contrast sensitivity under dim light, and the ability to extract the motion signal from a noisy background, which affects motion contrast sensitivity under all luminance conditions. Surprisingly, the current study suggests that the temporal properties of early processes and the level of neural noise affecting motion contrast sensitivity are relatively spared with healthy aging.

## Supplementary information

Supplementary Information.

## Data Availability

All data generated or analysed during this study are included in this published article (and its Supplementary Information files).
