# Supplementary material for: Age-related decline in motion contrast sensitivity due to lower absorption rate of cones and calculation efficiency
Source: Sci Rep. 2020 Oct 5;10:16521. doi: 10.1038/s41598-020-73322-7 (PMC7536415; doi:10.1038/s41598-020-73322-7)
Supplement: Supplementary file 1 — Supplementary Information. [file 41598_2020_73322_MOESM1_ESM.pdf]

Age-related decline in motion contrast sensitivity due to lower absorption rate of cones and calculation efficiency

Asma Braham chaouche, Daphné Silvestre, Arthur Trognon, Angelo Arleo, and Rémy Allard

Supplementary Data Set: Contrast thresholds

	Experiment 1																					Experiment 2				
Lum (Td)	0.35	1.1	1.1	3.5		3.5	11	35		110	351											110				
TF (Hz)	3.75	0.9375	3.75	1.875		7.5	3.75	15		30	0.9375		1.875		3.75		7.5		15		30	0.9375	1.875	3.75	7.5	15
Noise	None	None	None	None	None	None	None	None	None	None	None	High	None	High	None	High	None	High	None	High	None	High	High	High	High	High
Bg color	Grey	Grey	Grey	Grey	Red	Grey	Grey	Grey	Red	Grey	Grey	Grey	Grey	Grey	Grey	Grey	Grey	Grey	Grey	Grey	Grey	Grey	Grey	Grey	Grey	Grey
Young1	0.0952	0.0445	0.0392	0.0134	0.0165	0.0415	0.0104	0.0308	0.0288	0.2028	0.0123	0.1037	0.0085	0.0897	0.0056	0.1014	0.0046	0.1085	0.0058	0.0970	0.0587					
Young2	0.1113	0.0752	0.0513	0.0290	0.0281	0.0507	0.0170	0.0389	0.0336	0.2681	0.0205	0.1585	0.0214	0.1418	0.0084	0.1402	0.0055	0.1676	0.0103	0.1466	0.1061	0.0897	0.0981	0.0949	0.0868	0.1097
Young3	0.1088	0.0515	0.0513	0.0157	0.0241	0.0332	0.0103	0.0281	0.0291	0.2051	0.0115	0.0710	0.0065	0.0759	0.0055	0.0928	0.0040	0.1254	0.0068	0.1752	0.0621	0.0742	0.0574	0.0726	0.0702	0.0664
Young4	0.0788	0.0333	0.0303	0.0155	0.0172	0.0251	0.0089	0.0225	0.0208	0.1403	0.0174	0.0970	0.0087	0.0938	0.0070	0.0887	0.0044	0.0970	0.0078	0.0928	0.0465					
Young5	0.1088	0.0326	0.0434	0.0176	0.0213	0.0278	0.0099	0.0318	0.0430	0.1961	0.0114	0.1014	0.0068	0.0877	0.0040	0.0868	0.0039	0.1268	0.0065	0.1533	0.0531	0.0887	0.0907	0.0694	0.0785	0.1226
Young6	0.1041	0.0538	0.0561	0.0232	0.0257	0.0401	0.0147	0.0278	0.0275	0.1569	0.0210	0.0959	0.0125	0.0918	0.0058	0.1061	0.0045	0.1097	0.0072	0.1122	0.0581	0.0949	0.0821	0.0759	0.0671	0.0970
Young7	0.1439	0.0431	0.0485	0.0215	0.0275	0.0567	0.0119	0.0679	0.0594	0.3426	0.0131	0.1482	0.0100	0.0970	0.0059	0.1226	0.0092	0.1122	0.0153	0.2215	0.0821	0.1122	0.1434	0.1026	0.0970	0.2738
Young8	0.1376	0.0804	0.0749	0.0375	0.0329	0.0573	0.0164	0.0465	0.0336	0.2193	0.0227	0.1297	0.0136	0.1341	0.0065	0.1434	0.0075	0.1434	0.0143	0.2142	0.0928	0.0992	0.0776	0.0821	0.0694	0.1434
Young9	0.1345	0.0284	0.0444	0.0126	0.0152	0.0307	0.0077	0.0288	0.0257	0.2931	0.0172	0.0992	0.0076	0.0803	0.0074	0.0949	0.0065	0.0949	0.0106	0.0776	0.1109	0.0949	0.0918	0.0642	0.0514	0.0635
Young10	0.0788	0.0279	0.0281	0.0116	0.0167	0.0419	0.0090	0.0348	0.0389	0.2193	0.0080	0.0858	0.0058	0.0868	0.0032	0.1026	0.0035	0.1516	0.0077	0.1297	0.0812	0.0959	0.0887	0.0877	0.0686	0.1026
Young11	0.0880	0.0435	0.0410	0.0166	0.0260	0.0469	0.0127	0.0402	0.0376	0.1876	0.0133	0.0858	0.0094	0.0907	0.0065	0.1122	0.0058	0.1585	0.0093	0.1326	0.0568	0.0718	0.0642	0.0710	0.0751	0.0918
Young12	0.1007	0.0538	0.0424	0.0217	0.0230	0.0464	0.0168	0.0508	0.0435	0.2507	0.0232	0.0686	0.0140	0.0918	0.0093	0.0830	0.0069	0.1049	0.0162	0.1733	0.0877	0.0830	0.0768	0.0555	0.0574	0.1550
Young13	0.0745	0.0276	0.0307	0.0129	0.0144	0.0336	0.0072	0.0333	0.0420	0.2452	0.0074	0.0981	0.0028	0.0970	0.0028	0.1109	0.0022	0.1482	0.0052	0.1916	0.0389	0.1014	0.0877	0.0726	0.0702	0.1173
Young14	0.1231	0.0622	0.0641	0.0246	0.0389	0.0469	0.0196	0.0440	0.0475	0.2931	0.0159	0.1311	0.0098	0.1037	0.0062	0.1213	0.0051	0.1226	0.0107	0.1938	0.1003					
Young15	0.1029	0.0431	0.0507	0.0217	0.0249	0.0573	0.0114	0.0475	0.0531	0.5007	0.0140	0.1386	0.0080	0.1326	0.0067	0.1134	0.0044	0.1240	0.0108	0.1254	0.0794	0.0887	0.0776	0.0949	0.0918	0.1134
Young16	0.0659	0.0333	0.0424	0.0120	0.0184	0.0496	0.0433	0.0503	0.0336	0.2051	0.0089	0.0718	0.0058	0.0519	0.0051	0.0803	0.0055	0.1097	0.0147	0.1311	0.2590	0.0594	0.0465	0.0657	0.0803	0.0785
Young17	0.0589	0.0241	0.0328	0.0075	0.0144	0.0254	0.0074	0.0220	0.0301	0.1372	0.0051	0.0635	0.0032	0.0710	0.0026	0.0587	0.0025	0.0568	0.0050	0.0594	0.0502	0.0671	0.0574	0.0491	0.0562	0.0858
Young18	0.1164	0.0711	0.0573	0.0269	0.0266	0.0586	0.0164	0.0635	0.0503	0.3351	0.0194	0.1356	0.0107	0.1213	0.0102	0.1341	0.0073	0.1832	0.0147	0.1550	0.1341	0.1297	0.0959	0.1073	0.1109	0.1695
Young19	0.0995	0.0498	0.0474	0.0196	0.0225	0.0448	0.0139	0.0385	0.0380	0.2931	0.0245	0.0830	0.0139	0.0949	0.0092	0.0992	0.0059	0.1173	0.0113	0.1085	0.0928	0.0959	0.0887	0.0877	0.0686	0.1026
Young20	0.1391	0.0575	0.0507	0.0388	0.1327	0.0810	0.0251	0.0672	0.0830	0.4188	0.0296	0.1122	0.0150	0.0907	0.0075	0.0970	0.0071	0.0877	0.0194	0.1772	0.1254	0.0785	0.1122	0.1085	0.0803	0.1516
Old1	0.3632	0.0515	0.0655	0.0225	0.0269	0.0725	0.0190	0.0794	0.0687	0.5474	0.0091	0.1037	0.0070	0.0897	0.0064	0.0858	0.0061	0.0897	0.0177	0.1639	0.3385					
Old2	0.2628	0.2654	0.1850	0.0867	0.0702	0.1309	0.0248	0.0993	0.0939	0.2621	0.0254	0.1109	0.0203	0.0868	0.0075	0.0907	0.0069	0.0992	0.0171	0.1418	0.3096					
Old3	0.1301	0.0860	0.0968	0.0406	0.0440	0.0801	0.0259	0.0695	0.0601	0.3664	0.0317	0.1499	0.0108	0.0759	0.0075	0.0821	0.0095	0.1311	0.0232	0.2026	0.1499					
Old4	0.1125	0.0804	0.0693	0.0449	0.0526	0.1107	0.0317	0.1073	0.0982	0.6121	0.0362	0.1402	0.0166	0.1109	0.0122	0.1226	0.0088	0.1199	0.0177	0.1434	0.1356					
Old5	0.2810	0.1816	0.1480	0.0701	0.0687	0.1582	0.0324	0.1327	0.1227	0.6843	0.0321	0.1585	0.0256	0.1240	0.0111	0.1026	0.0095	0.1085	0.0256	0.1812	0.3310					
Old6	0.4341	0.1453	0.1353	0.0513	0.0455	0.1252	0.0331	0.1085	0.0939		0.0379	0.2395	0.0253	0.1482	0.0110	0.1240	0.0080	0.1240	0.0183	0.1567	0.2316					
Old7	0.2056	0.1257	0.1107	0.0561	0.0450	0.0886	0.0296	0.0908	0.0803		0.0186	0.1109	0.0128	0.0812	0.0094	0.0907	0.0090	0.1122	0.0259	0.1639	0.2831					
Old8	0.1923	0.0889	0.1211	0.0375	0.0385	0.0896	0.0194	0.0849	0.0759	0.4429	0.0196	0.1450	0.0105	0.1226	0.0067	0.1085	0.0071	0.1639	0.0130	0.1981	0.1199					
Old9	0.2298	0.1737	0.1530	0.0613	0.0385	0.1431	0.0468	0.1586	0.0830		0.0300	0.1874	0.0091	0.1621	0.0131	0.1297	0.0081	0.1402	0.0162	0.2265	0.2532					
Old10	0.3177	0.0193	0.0182	0.0272	0.0385	0.0947	0.0203	0.0679	0.0614	0.4429	0.0134	0.0949	0.0100	0.0794	0.0080	0.1073	0.0064	0.1122	0.0140	0.1482	0.1402					
Old11	0.1439	0.0503	0.0599	0.0164	0.0193	0.0424	0.0102	0.0411	0.0372	0.2898	0.0085	0.0600	0.0059	0.0657	0.0041	0.0679	0.0033	0.0868	0.0083	0.1213	0.1037					
Old12	0.1316	0.0899	0.1024	0.0406	0.0928	0.2187	0.0383	0.1813	0.1500		0.0094	0.0734	0.0056	0.0642	0.0045	0.0702	0.0051	0.1297	0.0121	0.1874	0.1695					
Old13	0.1901	0.1136	0.1047	0.0593	0.0481	0.0968	0.0347	0.1534	0.0868		0.0375	0.1341	0.0396	0.0992	0.0157	0.1533	0.0103	0.1482	0.0342	0.2590						
Old14	0.1759	0.0909	0.0958	0.0469	0.0344	0.1399	0.0321	0.1297	0.1026	0.5854	0.0254	0.1173	0.0177	0.1085	0.0082	0.1173	0.0057	0.1297	0.0143	0.2800	0.2291					
Old15	0.2056	0.1136	0.1107	0.0519	0.0726	0.1013	0.0310	0.1342	0.1148		0.0287	0.2049	0.0171	0.1122	0.0172	0.1466	0.0147	0.1371	0.0303	0.3273	0.3061					
Old16	0.1988	0.0822	0.0725	0.0246	0.0228	0.0542	0.0154	0.0679	0.0402	0.3746	0.0170	0.1297	0.0077	0.1186	0.0043	0.0949	0.0050	0.1003	0.0112	0.1450	0.1037					
Old17	0.1538	0.0615	0.0663	0.0336	0.0301	0.1071	0.0222	0.0803	0.0526	0.5177	0.0259	0.0812	0.0194	0.0821	0.0139	0.0726	0.0081	0.1226	0.0116	0.1450	0.1657					
Old18	0.2905	0.0727	0.0896	0.0263	0.0336	0.0709	0.0152	0.0726	0.0556	0.3961	0.0109	0.1061	0.0072	0.1147	0.0085	0.1085	0.0049	0.1418	0.0124	0.1695	0.1676					
Old19	0.1272	0.0575	0.0620	0.0230	0.0254	0.0627	0.0198	0.0734	0.0588	0.3623	0.0105	0.0897	0.0056	0.0830	0.0053	0.0970	0.0038	0.0959	0.0125	0.1311	0.1341					
